# Higher free triiodothyronine concentration is associated with lower prevalence of microangiopathic complications and better metabolic control in adult euthyroid people with type 1 diabetes

**DOI:** 10.1007/s12020-018-1582-8

**Published:** 2018-03-30

**Authors:** Bogusz Falkowski, Anita Rogowicz-Frontczak, Agata Grzelka, Aleksandra Uruska, Judyta Schlaffke, Aleksandra Araszkiewicz, Dorota Zozulinska-Ziolkiewicz

**Affiliations:** Department of Internal Medicine and Diabetology, Poznan University of Medical Sciences, Mickiewicza 2, Poznan, 60-834 Poland

**Keywords:** FT3, Free triiodothyronine, Type 1 diabetes mellitus, Microangiopathic complications, Euthyroid sick syndrome, Glycated hemoglobin

## Abstract

**Purpose:**

Type 1 diabetes mellitus (T1DM) is a disorder of insulin deficiency but with a wide range of hormones simultaneously disturbed. The study was performed to explore relation of free triiodothyronine (FT3) with metabolic control and occurrence of microangiopathic complications.

**Methods:**

A total of 266 adult T1DM participants [56% men; 32 (interquartile range, IQR: 25–39) years and disease duration 13 (IQR: 8–19) years] in euthyroid state with negative history for hypothyroidism were included to the study. Participants were screened for thyroid-stimulating hormone (TSH), free thyroxine (FT4) and FT3. Moreover, microangiopathic complications (retinopathy, diabetic kidney disease, peripheral and autonomic neuropathy), markers of metabolic control such as glycated hemoglobin (HbA_1c_) were evaluated.

**Results:**

A total of 114 (42.9%) people had diagnosed at least one microangiopathic complication. In multivariable linear regression higher HbA_1c_ was statistically significant independent predictor of lower FT3 (*β* = −0.25; *p* < 0.0001) after adjustment for sex, T1DM duration, HbA_1c_, waist-to-hip ratio (WHR) (*R*^2^ = 0.15, *p* < 0.0001). Higher FT3 was simultaneously a predictor of lower prevalence of microangiopathy in multivariate logistic regression analysis (odds ratio, 0.51; 95% confidence interval, 0.27–0.98; *p* = 0.04) after an adjustment for: age, hypertension, HbA_1c_, WHR and total cholesterol (TC).

**Conclusions:**

FT3 as tissue active hormone plays a clinically important role in T1DM people. The higher FT3 concentration is related to the lower prevalence of microangiopathy and better metabolic control of the disease in adult euthyroid people with T1DM.

## Introduction

Thyroid hormones are crucial for energy homeostasis and regulation of metabolism pace. They affect metabolism both centrally in connection with the hypothalamus and peripherally through key targets, such as the brown and white adipose tissue, the liver, muscles and pancreatic β-cells. Almost every major biochemical process, related to energy distribution in the human organism, such as lipogenesis, lipolysis, gluconeogenesis, glucose handling, insulin resistance, is affected by thyroid hormones [1].

In the current literature, there are not a lot of studies assessing thyroid impact on clinical features in the course of type 1 diabetes mellitus (T1DM). Lower total triiodothyronine (T3) concentrations were found in the group of 30 T1DM children as compared with 30 healthy controls [2]. The phenomenon of transient disorder of thyroid function is generally described as an euthyroid sick syndrome or non-thyroidal illness syndrome or low T3 syndrome [3]. This state is connected with acute or chronic disorders of various organs and systems and is characterized by total T3 and free triiodothyronine (FT3) concentrations below and thyroid-stimulating hormone (TSH) concentration within normal range. A previously conducted study in T1DM patients with diabetic ketoacidosis (DKA) was focused on demonstration of temporary and slight changes of total T3 [4]. Only a few studies were focused on thyroid function and microvascular complications of diabetes. However, in mentioned studies higher TSH was related to higher presence of diabetic kidney disease or retinopathy. FT3 was not assessed and mainly type 2 diabetes mellitus (T2DM) people were enrolled. Additionally, examined populations contained both euthyroid and hypothyroid participants [5–8].

The aim of the study was to assess the relation between FT3 concentration and metabolic control and occurrence of chronic microangiopathic complications in euthyroid T1DM people with negative history for hypothyroidism.

## Materials and methods

### Participants

The study was carried out in a T1DM adult population within a single center with duration of the disease equal to or above 5 years in the Department of Internal Medicine and Diabetology. Participants were collected between years 2013–2017. Diabetes was diagnosed based on American Diabetes Association criteria, which included: classic symptoms of hyperglycemia or hyperglycaemic crisis as well as random plasma glucose concentration of ≥11.1 mmol/L [9]. To confirm the evidence of autoimmune etiology at least one out of three examined autoantibodies (against islet cells [ICA], glutamic acid decarboxylase [GAD], insulinoma-associated tyrosine phosphatase [IA-2]) had to be positive.

333 T1DM participants with at least 5-year history of the disease were initially assessed. Following participants were excluded from the study: with hypothyroidism in history, with TSH actual concentration without normal range (normal range: 0.27–4.2 µIU/mL), with estimated glomerular filtration rate (eGFR) below 30 mL min^−1^ × 1.73 m^−2^, with alanine transaminase (ALT) or aspartate transaminase (AST) three times above normal range, with DKA or ketonuria at the time of enrollment to the study and anemia (hemoglobin concentration below 6.8 mmol/L). Participants taking drugs affecting glucose metabolism, anti-thyroid drugs, drugs affecting thyroid function (L-thyroxin, glucocorticosteroids, propranolol, drugs containing iodine, salicylates, phenytoin, phenobarbital, carbamazepine) were also excluded from the study [10]. Totally 67 participants were excluded due to exclusion criteria (Table 1). Owing to above-mentioned exclusion criteria, the study population comprised 266 participants including 149 (56.0%) men and 117 (44.0%) women. Median participants' age was 32 (interquartile range [IQR]: 25–39) years and T1DM duration was 13 (IQR: 8–19) years. Every participant was European Caucasian in origin.

**Table 1:** Inclusion and exclusion criteria

Inclusion criteria	• Type 1 diabetes • Age above 18 years • Duration of the disease ≥5 years
Exclusion criteria	• Hypothyroidism in history • TSH actual concentration without reference interval • eGFR below 30 mL min^−1^ × 1.73 m^−2^ • ALT or AST three times above reference interval • With DKA or ketonuria at the time of enrollment to the study • Anemia • Taking drugs affecting glucose metabolism, anti-thyroid drugs, drugs affecting thyroid function: L-thyroxin, glucocorticosteroids, propranolol, drugs containing iodine, salicylates, phenytoin, phenobarbital, carbamazepine

*ALT* alanine transaminase, *AST* aspartate transaminase, *DKA* diabetic ketoacidosis, *eGFR* estimated glomerular filtration rate, *TSH* thyroid-stimulating hormone

The study followed the Declaration of Helsinki guidelines concerning research conducted on human subjects. Bioethical Committee approved the study protocol.

### Assessment of microangiopathy

Direct ophthalmoscopy through dilated pupils was applied to assess diabetic retinopathy presence. Results of examination were classified as no retinopathy, mild non-proliferative, moderate non-proliferative, severe non-proliferative and proliferative retinopathy according to the classification of the American Academy of Ophthalmology [11].

To diagnose diabetic kidney disease urinary albumin excretion was collected for 24-h or random albumin/creatinine ratio expressed in milligrams of albumin per gram of creatinine (mg/g) was evaluated. Excretion rate more than 30 mg/24 h or 30 mg/g at least in two samples collected over 3 months enabled us to diagnose persistent albuminuria. Any conditions which could interfere evaluation of urinary albumin excretion were excluded. The secondary causes of proteinuria were taken into consideration: excessive physical activity, acute febrile illness, urinary tract infection, heart failure and hematuria. For diagnosis of diabetic kidney disease persistent albuminuria and over 10 years diabetes duration time or confirmed diabetic retinopathy were required [12].

Peripheral neuropathy was assessed by 10 g monofilament examining pressure perception, 128 Hz tuning fork to evaluate vibration perception and additionally ankle reflex tests. Following components were taken into consideration: symptoms of neuropathy reported by participants, abnormal result of ankle reflex tests, abnormal pressure and/or vibration perception. Two or more of the above-mentioned were required to make a diagnosis of diabetic neuropathy [13].

Evaluation of cardiovascular autonomic neuropathy was based on set of tests including: resting tachycardia assessment (defined as heart rate over 100 beats per minute), orthostatic hypotension presence (diagnosed in standing position as compared with recumbence when the drop in systolic blood pressure [SBP] and/or diastolic blood pressure [DBP] was at least 20 mmHg and 10 mmHg, respectively), heart rate variability assessed using PROSCICARD III device (CPS MEDICAL, USA, 2010) [14].

Retinopathy, peripheral and cardiac autonomic neuropathy and diabetic kidney disease were considered as microvascular complications. Microangiopathy was assessed as present in the case of at least one microvascular complication of diabetes found in examination. These participants were qualified to the group with microangiopathy.

### FT3, FT4, TSH and HbA_1c_ measurement

Venous blood samples were collected between 8:00 and 10:00 am by the S-Monovette blood collection system (Sarstedt, Aktiengesellschaft & Co, Numbrecht, Germany) after at least 12-h overnight fasting using a standard venipuncture technique. Serum was obtained after clotting at room temperature and centrifuging at 2000×*g* for 15 min. Laboratory analysis was performed in the day when blood was taken.

Electrochemiluminescence immunoassay (ECLIA) Elecsys TSH, Elecsys FT4 and Elecsys FT3 on Cobas analyzers (Roche Diagnostics, Basel, Switzerland) were applied to measure TSH, FT4 and FT3. ECLIA method uses monoclonal antibodies directed against the human proteins. The TSH measuring range was between 0.005 and 100.0 µIU/mL. Reference interval of TSH was 0,27–4,2 µIU/mL. The FT4 measuring range was between 0.5 and 100.0 pmol/L. Reference interval of FT4 values for adults was 12–22 pmol/L. The FT3 measuring range was between 0.4 and 50.0 pmol/L. Reference interval of FT3 values for adults was 3.1–6.8 pmol/L.

HbA_1c_ was measured in venous blood with competitive turbidimetric inhibition immunoassay method on Cobas analyzer (Roche Diagnostics, Basel, Switzerland) and expressed as g/dL. The HbA_1c_ measuring range was between 0.186 and 1.61 g/dL. The Twin Test reaction mode was used and it allows sequential measurement of the hemoglobin and HbA_1c_ in a single cuvette. Final result expressed in IFCC units (mmol/mol) and equivalent in % were calculated from the HbA_1c_/hemoglobin concentration according to DCCT/NGSP (IFCC, The International Federation of Clinical Chemistry and Laboratory Medicine; DCCT, Diabetes Control and Complication Trial; NGSP, National Glycohemoglobin Standardisation Program).

### Assessment of other variables

Every participant underwent comprehensive physical examination including anthropometric measurements and blood pressure evaluation. Body mass index (BMI) was obtained by dividing participants weight (expressed in kilograms) by squared height (in meters). Participant’s height and weight were evaluated using standard methods. WHR was obtained by dividing waist and hips circumferences. Waist and hips circumferences were measured in standing position at the umbilical and greater trochanters of the femurs levels, respectively. Hypertension was assessed according to participant’s history or was newly diagnosed. Blood pressure was measured in a sitting position by the oscillometric method after at least 10 min rest. New diagnosis was made after two measurements in two different days in the case of mean values of these two measurements over 140 mmHg (SBP) and/or 90 mmHg (DBP).

High-density lipoprotein (HDL), low-density lipoprotein (LDL), triglycerides (TG), total cholesterol (TC), C-reactive protein (hsCRP), ALT and AST were measured using commercially available enzymatic assays (Roche Diagnostics, Basel, Switzerland). eGFR was calculated using The Chronic Kidney Disease Epidemiology Collaboration equation [9, 15]. Following parameters were inserted into the equation: creatinine concentration in serum, age and sex.

Additionally, some information (age, T1DM duration, sex, hyper/hypothyroidism, smoking) was received via a questionnaire survey. Patient was qualified as smoker where smoking was actually declared in the moment of questionnaire completing.

### Statistical analysis

All statistical analyses were performed using the commercially available software STATISTICA V13 PL (Statsoft, Tusla, Oklahoma, United States). Shapiro–Wilk test was applied to assess distributions of continuous variables. Distributions of all the continuous variables (except WHR) were not normal. Data are presented in the form of the number (%) for categorical variables or median (IQR) for continuous variables. For baseline characteristics analysis, the differences between variables measured on dichotomous scales (sex, smoking and presence of hypertension) were compared using *χ*^2^ test and Mann–Whitney *U* test for continuous variables (Table 2). Correlations between FT3 and various laboratory and clinical findings were assessed by means of Spearman’s rank correlation analysis (Table 3). We correlated only factors potentially affecting FT3 due to literature. Multivariate linear regression analysis was used to examine relationships of FT3 and HbA_1c_ because of adequate number of participants to use this method (Table 4). Predictors were chosen based on statistically significant correlations (Table 3) and criteria described under Table 4. Results are presented in the form of estimated effects (*β*) with appropriate *p-*value for predictors and coefficient of determination (*R*^2^) with appropriate *p*-value for the whole model. The multiple logistic regression model was used to examine the relationships between the prevalence of microangiopathic complications in T1DM people and concentration of FT3. Predictors were chosen based on presented in Table 2 baseline characteristics and described under Table 4 exclusions. Odds ratios (ORs) per unit with their corresponding 95% confidence intervals (CIs) are presented. A *p*-value of less than 0.05 was considered statistically significant in every analysis.

**Table 2:** Characteristics of the study participants with and without microangiopathy. Data are presented in the form of number (%) or median (IQR)

Parameter	Value			
	All participants (*n* = 266)	With microangiopathy (*n* = 114)	Without microangiopathy (*n* = 152)	p*
Age, years	32 (25–39)	38 (29–47)	29 (24–36)	< **0.0001**
Men, *n* (%)	149 (56.0)	65 (57.0)	84 (55.3)	0.77^a^
Smoker, *n* (%)	63 (23.7)	28 (24.6)	35 (23.0)	0.77^a^
T1DM duration, years	13 (8–19)	18 (14–28)	9.5 (7–13)	< **0.0001**
Hypertension, *n* (%)	70 (26.3)	49 (43.0)	21 (13.8)	< **0.0001**^a^
BMI (kg/m^2^)	24.76 (22.32–27.47)	25.54 (22.59–28.07)	24.19 (22.10–26.66)	0.07
WHR	0.86 (0.79–0.92)	0.88 (0.80–0.93)	0.83 (0.78–0.91)	**0.005**
HbA_1c_ (mmol/mol)	61 (52–72)	63 (54–73)	57 (50–70)	**0.02**
HbA_1c_ (%)	7.7 (6.9–8.7)	7.95 (7.1–8.8)	7.4 (6.7–8.6)	**0.02**
hsCRP (mg/L)	1.07 (0.50–2.61)	1.13 (0.50–2.91)	1.04 (0.51–2.19)	0.60
ALT (U/L)	17 (13–22)	19 (14–26)	15 (12–20)	**0.0008**
AST (U/L)	17 (15–21)	19 (15–23.5)	17 (14–19.5)	**0.0007**
TC (mmol/L)	4.92 (4.26–5.58)	5.02 (4.53–5.85)	4.73 (4.09–5.44)	**0.0001**
HDL (mmol/L)	1.68 (1.42–1.94)	1.73 (1.42–2.07)	1.66 (1.41–1.92)	0.13
LDL (mmol/L)	2.82 (2.28–3.57)	2.91 (2.43–3.65)	2.80 (2.18–3.44)	0.15
TG (mmol/L)	0.97 (0.73–1.39)	1.11 (0.84–1.63)	0.89 (0.68–1.20)	**0.0001**
eGFR (mL min^−1^ × 1.73 m^−2^)	106.32 (94.19–117.59)	98.21 (86.51–113.49)	110.32 (99.71–118.75)	< **0.0001**
TSH (µIU/mL)	1.65 (1.19–2.13)	1.58 (1.11–2.11)	1.72 (1.28–2.17)	0.17
FT4, pmol/L**	15.19 (13.90–16.73)	15.45 (14.16–16.86)	14.80 (13.77–16.60)	0.23
FT3, pmol/L	4.79 (4.31–5.24)	4.69 (4.29–5.07)	4.84 (4.38–5.33)	**0.04**

*p-value for comparison groups in accordance to presence of microangiopathy

**125 participants had FT4 assessed (34 with microangiopathy, 91 without microangiopathy)

^a^*χ*^2^ test; Mann–Whitney *U* test in every other case, where it is not marked with the letter

*ALT* alanine transaminase, *AST* aspartate transaminase, *BMI* body mass index, *FT3* free triiodothyronine, *FT4* free thyroxine, *eGFR* estimated glomerular filtration rate, *HbA*_*1c*_ glycated hemoglobin, *HDL* high-density lipoprotein, *hsCRP* high sensitive C-reactive protein, *IQR* interquartile range, *LDL* low-density lipoprotein, *T1DM* type 1 diabetes, *TC* total cholesterol, *TG* triglycerides, *TSH* thyroid-stimulating hormone, WHR waist-to-hip ratio

Bold *p*-values are statistically significant (a *p*-value less than 0.05)

**Table 3:** Correlations between various parameters and FT3 concentration in blood (Spearman’s rank correlation analysis)

Parameter	*r*	*p*-value
Age	−0.05	0.43
T1DM duration	−**0.17**	**0.006**
BMI	**0.14**	**0.02**
WHR	**0.23**	**0.0002**
HbA_1c_	−**0.31**	**<0.0001**
hsCRP	−0.06	0.34
TC	−0.02	0.71
HDL	−0.11	0.06
LDL	0.06	0.36
TG	0.02	0.80
eGFR	0.11	0.06
TSH	−0.01	0.82
FT4	**0.38**	**<0.0001**

Bold *p*-values are statistically significant (a *p*-value less than 0.05)

Abbreviations: see Table 2

**Table 4:** Predictors of FT3 in multivariate linear regression analysis with FT3 as dependent variable and sex, T1DM duration, HbA_1c_, WHR as independent variables. Model performance: *R*^2^ = 0.15, *p* < 0.0001

Predictors	Multivariate analysis	
	*β*	*p*-value
Male sex	0.16	**0.02**
T1DM duration	−0.15	**0.01**
HbA_1c_	−0.25	**<0.0001**
WHR	0.08	0.22

Following predictors were taken into account: T1DM duration, TSH, FT4, BMI, WHR, HbA_1c_ and sex. Predictors were chosen based primarily on statistically significant correlations (Table 3). Sex was inserted into regression based on widely known dependence of hypothyroidism on sex. BMI was not inserted into the analysis because of significant correlation with WHR and WHR was chosen because this predictor was better correlated with FT3. FT4 was inserted into second regression model (Table 5) to assess predictors from this model with full model. TSH was not inserted into analysis because significant correlation with FT3 did not occur

Bold *p*-values are statistically significant (a *p*-value less than 0.05)

Abbreviations: see Table 2

## Results

In the study group, 114 (42.9%) participants were diagnosed with at least one of microangiopathic complications. The prevalence of diabetic kidney disease was 12.4%, retinopathy 37%, autonomic neuropathy 13.9% and peripheral neuropathy 17.7%. At the time of enrollment, all participants were euthyroid and had negative history for hypothyroidism. FT3 but neither TSH nor FT4 concentrations were statistically significantly different in the group of participants with microangiopathy as compared with participants without these complications [median 4.69 (IQR: 4.29–5.07) vs. median 4.84 (IQR: 4.38–5.33) pmol/L respectively; *p* = 0.04] (Table 2). According to expectations, groups differed statistically significantly with respect to age, T1DM duration, hypertension prevalence and eGFR. Other differences are presented in Table 2.

Statistically significant positive correlations between FT3 and: WHR (*r* = 0.23; *p* = 0.0002), BMI (*r* = 0.14; *p* = 0.02) and FT4 (*r* = 0.38; *p* < 0.0001) were found. Moreover, FT3 correlated negatively with HbA_1c_ (*r* = −0.31; *p* < 0.0001) and T1DM duration (*r* = −0.17; *p* = 0.006) (Table 3). Additionally, in multivariate linear regression analysis higher HbA_1c_ turned out to be statistically significant independent predictor of lower FT3 (*β* = −0.25; *p* < 0.0001) after adjustment for sex, T1DM duration and WHR (*R*^2^ = 0.15, *p* < 0.0001) (Table 4). Multivariate linear regression analysis extended to include FT4 was also conducted. Following second analysis, higher HbA_1c_ remained statistically significant independent predictor of lower FT3 (*β* = −0.38; *p* < 0.0001) after adjustment for sex, T1DM duration, WHR and FT4 (*R*^2^ = 0.24, *p* < 0.0001) (Table 5). Moreover, HbA_1c_ as compared with FT4 turned out to be stronger predictor of FT3.

**Table 5:** Predictors of FT3 in multivariate linear regression analysis with FT3 as dependent variable and sex, T1DM duration, HbA_1c_, WHR and FT4 as independent variables. Model performance: *R*^2^ = 0.24, *p* < 0.0001

Predictors	Multivariate analysis	
	*β*	*p*-value
Male sex	0.11	0.29
T1DM duration	−0.16	0.05
HbA_1c_	−0.38	**<0.0001**
WHR	0.05	0.62
FT4	0.19	**0.02**

Reasons why some predictors were excluded are described under Table 4. FT4 was added to analysis to assess significance of dependence presented in Table 4 in the light of widely known relation FT4 to FT3

Bold *p*-values are statistically significant (a *p*-value less than 0.05)

Abbreviations: see Table 2

Higher FT3 was a predictor of lower prevalence of microangiopathy in multivariate logistic regression analysis [OR, 0.51; 95% CI, 0.27–0.98; *p* = 0.04] after an adjustment for potential explanatory factors: age, hypertension, HbA_1c_, WHR and TC (Table 6).

**Table 6:** Predictors of microangiopathy in multivariate logistic regression analysis with microangiopathy presence as dependent variable and age, hypertension, HbA_1c_, WHR, TC and FT3 as independent variables (*p* < 0.0001)

Predictors	Odds ratio (95% confidence interwal)	*p*-value
Age	1.07 (1.04–1.11)	**<0.0001**
Hypertension	2.69 (1.34–5.39)	**0.005**
HbA_1c_	1.24 (1.01–1.53)	**0.04**
WHR	7.20 (0.33–158.38)	0.21
TC	1.25 (0.93–1.67)	0.14
FT3	0.51 (0.27–0.98)	**0.04**

Predictors were chosen based on presented in Table 2 baseline characteristics. Following predictors were taken into account: age, T1D duration, hypertension, WHR, HbA_1c_, ALT, AST, TC, TG, eGFR and FT3. Age was chosen arbitrally and subsequently T1D duration was excluded as closely related to patients' age. ALT and AST were not inserted into the analysis because they are different between group with microvascular complications vs. group without microvascular complications as an effect of diabetic hepatopathy. Lower eGFR in a group with microvascular complications is not a risk factor of microvascular complications but is an effect of diabetic kidney disease. TC was chosen and TG subsequently was excluded because these covariates are closely interrelated

Bold *p*-values are statistically significant (a *p*-value less than 0.05)

Abbreviations: see Table 2

## Discussion

The most important result obtained in this study was a higher concentration of FT3 in euthyroid T1DM participants without presence of microvascular complications in comparison to the participants with microangiopathy. Another important finding is also an inverse correlation of FT3 and current metabolic control of diabetes.

Low FT3 in relation to euthyroid sick syndrome phenomenon has been shown in the group of DKA patients. DKA is an acute state of imbalanced blood gases and concentration of glycemia resulted from absolute deficiency of insulin. What is interesting, study conducted on DKA patients, showed that concentration of total T3 and FT3 were significantly lower during admission to the ward and became normal soon until patients were discharged [3].

Chronic state of poor glycemic control can lead to lower FT3 concentration as well. Similar observation to our study was presented in cohort of people with diabetes. Population was characterized by lower total T3 concentrations in the course of worse disease control in the form of higher HbA_1c_ and unexpectedly lower relative body weight (indicator calculated in this study as body weight to standard weight ratio) [16]. Although the results are interesting, methods are not acknowledged at present because of unclear type of diabetes among enrolled participants. Additionally, total T3 concentrations measurement is not widely in use. Moreover, correlation between total T3 and worse metabolic control was obtained on 64 T1DM but hypothyroid children [17]. Besides, there is no study excluding people with overt or subclinical thyroid disorders which influence metabolic control of diabetes and FT3 concentration [17]. The impact of diabetes and its complications on thyroid hormones concentrations should be assessed in the cohort of participants without important confounding factors as overt thyroid disease.

Although, the effect of poor metabolic control on FT3 in T1DM people without DKA is not as spectacular as in the case of DKA it is still significant what was presented in the current study. Higher FT3 concentration can be an effect of adequate control of diabetes in the form of desired HbA_1c_ concentration. Moreover, this state can be caused by changes in expression of glucose transporters (GLUTs) on the cell surface. GLUTs are responsible for transporting glucose into cells. Basal expression of GLUT is stimulated by thyroid hormones [1]. Thus, higher FT3 in the course of chronic disease like T1DM can also decrease glycemia and improve metabolic control. Although above mentioned processes are closely related to regulation of glycemia, to the best of our knowledge there is no comprehensive study concerned on impact of thyroid hormones on HbA_1c_ conducted on great group of euthyroid T1DM people with negative history for thyroid disorders. Current study is the first conducted to assess pure impact of thyroid hormones on metabolic control of the disease.

It was proved that development of microvascular complications of diabetes is closely related to metabolic control of diabetes. Chronic hyperglycemia enhances oxidative stress and in turn decreases FT3, what was observed in current study. Oxidative stress and proinflammatory cytokines increases deiodinase type III activity in tissues what effects in lower total T3 concentration but increases reverse triiodothyronine concentration [18]. Furthermore, excessive cytokine release observed in diabetes could be responsible for lower T4 uptake in peripheral tissues and T4 binding to proteins. Lower concentration of T4 in tissues converting it to T3 may lower total T3 concentration. Probably, it is an effect of energy-saving mechanism in situation of chronic disease. In contrast to theoretical predictions, HbA_1c_ as compared with FT4 turned out to be stronger predictor of FT3. Moreover, altered TSH glycosylation is very probable to play an important role in the case of chronic hyperglycemia. In effect, it could decrease TSH biological activity [19]. However, the findings of the current study do not support theory about important TSH role in the process of microangiopathy development.

To our knowledge this is the first performed study related to the occurrence of microvascular (currently also called neurovascular) complications and FT3 concentration along euthyroid people. Endothelial dysfunction is tightly connected with microvascular complications of diabetes [20]. It is known that hypothyroid people have reduced responsiveness to sublingual nitroglycerin as endothelium-independent stimulus as well as endothelium-dependent stimulus in the form of flow mediated dilatation [21]. Moreover, elevated TSH concentration is responsible for attenuating expression of endothelial nitric oxide synthase and prostacyclin [22]. Thus, the influence of thyroid status on endothelium is verified but studies on euthyroid populations are still lacking [23]. Perhaps the adaptive mechanisms in the form of low T3 in response to hyperglycemia in the chronic state lead to a vicious circle mechanism resulting in further impairment of the function of the endothelium. On the other hand, lower oxidative stress and undisturbed endothelial function may be related to higher FT3 in people with T1DM uncomplicated by microangiopathy.

Although the association of thyroid hormones with the function of the endothelium is known, the only one study focusing on this subject has been conducted on T1DM cohort group before [7]. The results of that study showed that lower TSH concentrations (0.4–2.5 mU/L) were associated with a lower risk of diabetic retinopathy and renal dysfunction in the form of lower eGFR, but TSH relation to the presence of diabetic kidney disease was not statistically significant. Unfortunately, this study assessed only TSH, FT3 was not measured, and was based on ethnically diverse cohort of participants. Additionally, few researches were focused on T2DM people [5, 6, 8]. Yang et al. found that sight-threatening diabetic retinopathy occurred more frequently in people with higher TSH as compared with group of lower TSH also in euthyroid population. Remaining studies were focused on hypothyroid population. It was revealed that people with higher TSH values had higher prevalence of diabetic kidney disease [5]. Connecting described interrelationships, we can explain why T1DM people with higher FT3 are less prone to microangiopathy and conversely, why T1DM people without microangiopathy are more prone to have higher FT3.

In our study in euthyroid T1DM participants, higher FT3 concentration correlated positively with markers of obesity such as BMI and WHR. Similar results were achieved in euthyroid adults without diabetes in the National Health and Nutrition Examination Survey 2007–2008 in the USA [24]. Study conducted on euthyroid participants without diabetes showed a positive correlation of FT3 and insulin resistance in the form of higher HOMA-IR (homeostatic model assessment-insulin resistance) score [25]. Unfortunately, people with diabetes were excluded from this study. It is very probable that increased concentration of FT3 is a result of increased resistance to thyroid hormones in peripheral tissues in obesity [25]. Moreover, it is known that obese people have higher concentrations of leptin what is an effect of peripheral leptin resistance. The increase of leptin concentrations stimulates excreting thyrotrophin-releasing hormone (TRH) from the hypothalamus [26]. Additionally, leptin promotes conversion T4 to T3 via escalating of the activity of deiodinases [27]. What is more, T1DM people are characterized by higher leptin concentrations in comparison with healthy controls independently of obesity [28].

Some limitations of our study should be emphasized. They are a single-center outreach, single time point of FT3 evaluation. Moreover, antithyroid autoantibodies (TPOAb and TGAb) were not assessed, but participants with hypothyroidism in history or TSH actual concentration out of the reference interval were excluded. Besides, we cannot fully explain relationship between lower FT3 and microvascular complications in the course of this research. However, it is certain that thyroid influence on clinical aspects of T1DM requires more comprehensive and precise research.

To conclude, FT3 as tissue active hormone plays an certain role in clinical features of T1DM people. The higher FT3 concentration is related to the lower prevalence of microangiopathy and better metabolic control of the disease in adult euthyroid people with type 1 diabetes. Nevertheless, clinical significance of described interrelationships requires profound researches within specific group of patients.
